# Inequalities in maternal healthcare use in Sierra Leone: Evidence from the 2008–2019 Demographic and Health Surveys

**DOI:** 10.1371/journal.pone.0276102

**Published:** 2022-10-13

**Authors:** Mluleki Tsawe, A Sathiya Susuman

**Affiliations:** 1 Department of Population Studies and Demography, North-West University, Mahikeng, South Africa; 2 Faculty of Humanities, Population and Health Research Focus Area, North-West University, Mahikeng, South Africa; 3 Department of Statistics & Population Studies, University of the Western Cape, Cape Town, South Africa; Duke University, UNITED STATES

## Abstract

**Background:**

Sierra Leone is one of the countries with poor health outcomes. The country has made some progress in the uptake of maternal health services. Despite improvements in the national coverage rates, there is no evidence of how equal these improvements have been.

**Objective:**

To estimate inequalities in maternal healthcare use in Sierra Leone.

**Methods:**

Using cross-sectional study data from 2008, 2013, and 2019 Demographic and Health Surveys (DHS), we study inequalities in maternal health services by computing rate ratios, and concentration indices (as well as concentration curves) using selected inequality stratifiers such as household wealth index, maternal education, and rural/urban place of residence.

**Results:**

We found that considerable progress has been made in increasing the uptake of maternal health services as well as reducing inequalities over time. We also found that inequalities in the selected health indicators favoured women from wealthy households, educated women, as well as women from urban areas. Although inequalities declined over time, the use of delivery services was highly unequal. However, of the selected health interventions, the use of four or more antenatal visits was almost at perfect equality in 2013 and 2019.

**Conclusion:**

Although efforts have been made to increase the use of maternal healthcare services among women with a lower socioeconomic status, the use of maternal health services remains favourable to women with a higher socioeconomic standing. Therefore, policy initiatives need to prioritise women of lower socioeconomic status through projects aimed at increasing women’s educational levels as well as focusing on poverty reduction.

## Introduction

The improvement of women’s sexual and reproductive health and rights remains important in the fight to reduce child and maternal mortality. Low- and middle-income countries (LMIC), often have maternal mortality ratios (MMR) due to childbirth-related complications. Estimates show that the Sub-Saharan Africa region has a soaring MMR of 542 per 100000 live births [1]. Three countries made the largest contribution to the MMR in the region; these include South Sudan (1150 maternal deaths per 100000 live births), Chad (1140 maternal deaths per 100000 live births) and Sierra Leone (1120 maternal deaths per 100000 live births) [1]. These high rates are partially due to various challenges which tend to intensify them such as lack of access to and provision of healthcare services, lack of or inadequate use of family planning services, malnutrition, and other issues [2–5]. Many countries have made significant strides toward meeting their Millennium Development Goals (MDG) targets, especially when it comes to the use of maternal health services [6]. However, the progress made at the national level tends to hide the inequalities that still exist at lower levels of geography (i.e., districts and chiefdoms). The level of maternal health service use differs between different socioeconomic groups within a country. It remains to be seen if the Sustainable Development Goals (SDGs) will build on the MDGs in increasing the use of maternal health services while reducing inequalities between socioeconomic groups.

Sierra Leone has had challenges trying to reduce the MMR and improve maternal health service provision. After years of struggles in dealing with high maternal mortality levels and poor uptake and provision of maternal health services (i.e., home birth deliveries) due to unaffordability, the government introduced the Free Health Care Initiative (FHCI) around 2010 as a way of improving the use of maternal and child healthcare [7,8]. In poor communities, out-of-pocket (OOP) expenditure on health becomes unrealistic. The FHCI removed user fees for women and young children needing to use healthcare services [9]. This led to some improvements in the update of life-saving maternal health services. The percentage of births that were delivered at home decreased over time in the country from 71.8% in 2008, 24.4% in 2013, and 16.4% in 2019 [10–12]. High financial costs often become a barrier to healthcare use, especially in rural areas where women are expected to travel long distance and pay more for transport to reach health services, especially in rural areas [13–15]. However, there is evidence of the existence of health inequalities in the country despite many improvements in maternal healthcare use. Studies show the existence of wealth-based health inequalities in some parts of the country [16,17].

To analyse inequalities in maternal healthcare, we adopted the framework developed by the Commission on Social Determinants of Health (CSDH). The CSDH framework argues that social position is an important determinant of health inequities [18]. The framework considers different elements of inequality such as socioeconomic status, education, race and geographic location as well as other elements [18–20]. In this study, we included maternal education, and household wealth index as the structural determinants of health; we also include the place of residence in the analysis. The structural determinants are part of the social and economic context of individuals and these are often regarded as the actual social determinants of health [18]. Inequalities in healthcare, especially in LMIC have drawn a lot of attention in recent times [21]. Addressing the health needs of the populations in lower socioeconomic positions is crucial in improving the overall health of the entire population. Although a few studies on healthcare inequalities have been conducted in Sierra Leone, many have focused on different aspects of healthcare inequality, and have used other measures and datasets, than those considered in this study [16,17,22,23]. This study aims to explore the extent of health inequalities in maternal healthcare as well as possible changes in these inequalities in Sierra Leone.

## Methods

### Data sources

We used cross-sectional data from the 2008, 2013, and 2019 Demographic and Health Surveys (SLDHS). The DHS collects nationally representative data on various health-related interventions. This data is publicly available for download upon request. The DHS data are among the widely used sources of data for analysis of health-related inequalities. The DHS conducted in Sierra Leone sampled 7 758 households in 2008, 13 006 households in 2013, and 13 793 households in 2019, with a response rate of 97.6%, 99.3%, and 99.5% respectively [10–12]. For all data collection points, women of reproductive age (WRA) who were either usual household members or women present in the household on the night before the survey were eligible for interviews. We indicate the study sample in Table 1.

**Table 1:** Sample used in the study.

Survey year	4+ ANC visits	Skilled ANC	Facility births	SBA
2008 SLDHS	3 380	4 103	5 651	5 811
2013 SLDHS	7 532	8 647	12 079	12 198
2019 SLDHS	6 448	7 326	9 771	9 771
**Total**	**17 361**	**20 076**	**27 501**	**27 781**

**Note**: The samples excluded “missing” and/or “do not know” cases.

## Maternal health indicators

This section presents the maternal health indicators used in the study. We selected the following indicators: (i) four or more antenatal visits, (ii) skilled antenatal care providers, (iii) births assisted by a skilled birth attendant (SBA), and (iv) births delivered in a facility. We defined four or more antenatal care visits as women who had at least four or more antenatal care visits for their most recent pregnancy; this definition has been used elsewhere [24,25]. We defined skilled antenatal care providers as women whose antenatal visits (for the most recent birth) were attended by a skilled provider. We defined births delivered in a facility as births that were delivered in a health facility; the health facilities included a government hospital, government health centre, government health post, other public sector, private hospital/clinic, and other private sector. We also defined births assisted by a skilled birth attendant (SBA) as births that were assisted by a skilled birth attendant (i.e. skilled provider). A skilled provider included a doctor, nurse/midwife, or auxiliary midwife. We dichotomised the selected indicator variables and we coded them as 0 = no and 1 = yes. **Inequality stratifiers and measures**

This study used three stratifiers to measure health-related inequality (maternal education, household wealth index, and place of residence). The household wealth index was computed for each household [using the Principal Components Analysis (PCA) method] to disaggregate the sample into equal-sized quintiles (i.e. poorest to richest) [26]. We measured the prevalence of four maternal health indicators for each of the data points considered in this study. We used rate ratios to measure absolute differences in inequalities using the selected inequality stratifiers. The rate ratios provide a general description of the extent of inequalities. The rate ratios for the wealth index were measured in terms of highest versus lowest household wealth quintile (RRhhwi = Rhighest quintile ÷ Rlowest quintile). The rate ratios for maternal education were measured in terms of highest versus none (RReduc = Rhigher ÷ Rnone). The rate ratios for residence were measured in terms of urban vs rural (RRur = Rurban ÷ Rrural). The main limitation of the measures above is that they provide a basic picture of inequalities and ignore the differentials that often exist between all the categories of the inequality stratifier. For instance, in computing the rate ratios for the household wealth index, only two extremes are considered (richest quintile to poorest quintile) and not the rest of the quintiles.

To remedy this, we used the concentration index. The concentration index is the most used measure of health inequalities in public health studies. It shows the magnitude of health-related inequalities and whether these inequalities are concentrated among those with low socioeconomic status or those with high socioeconomic status. The index value becomes negative when the health intervention is concentrated among the poor and positive when it is concentrated among the rich [27]. If the concentration index is negative, then the health indicator is said to be concentrated among individuals with low socioeconomic status, while a positive concentration index shows that the health indicator is concentrated among individuals with a high socioeconomic status [28]. Therefore, to further quantify inequalities in the selected indicators, we employed the concentration index. Specifically, we employed the Erreygers corrected concentration index.

$$E(h)=4\frac{\mu }{\left(b_{h}-a_{h}\right)}*C(h)$$

where *b_h_* and *a_h_* refer to the maximum and minimum bounds of the binary health indicator, *μ* refers to the mean of the health indicator, and *C*(*h*) refers to the concentration index [29]. The Erreygers corrected concentration index is recommended for use when the variable is binary [30]. The concentration curve is used to visualise the extent of inequalities in terms of the concentration index and the inequality stratifier is ranked across the x-axis and the cumulated fraction of the health intervention is plotted on the y-axis, and a diagonal line represents the line of equality [27]. Where the health intervention lies below the equity line, then it is said that there are pro-rich inequalities in that society, and when it lies above the equity line, then it is said there are pro-poor inequalities. We used the *conindex* command in Stata to estimate the corrected concentration index [31]. Various studies have used DHS data and applied similar methods to analyse the trends, determinants, and inequalities in maternal, child, and reproductive health interventions as well as service coverage [4,28,32–34].

### Complex samples

For all the data points, the SLDHS used a two-stage cluster sampling approach to select respondents for the surveys [10–12]. As such, we needed to adjust for data representation in our analysis; therefore, we used the Stata *svyset* command to account for the under- and over-sampling of certain enumeration areas. An alpha (α) level of 0.05 was considered statistically significant. We used Stata version 14.2 [35] and Microsoft Excel for all analyses of this study.

### Ethical considerations

We conducted all analyses using publicly available data from the SLDHS. The Institutional Review Board of Macro International, Inc. reviewed and approved the collection of data for all periods of the SLDHS data used in this study. Permission was granted to the authors by the DHS program to use this data for this study. For more information on the ethical review processes used by the DHS program. See more details on the ethical considerations in DHS data here: http://goo.gl/ny8T6X.

## Results

### Prevalence and rate ratios

Table 2 shows the ratios of education-related inequalities among WRA between those with no education and those with higher levels of education. There was an increase in the use of maternal health services over the three periods. The use of delivery care services (facility-based delivery and skilled birth attendance) doubled between 2008 and 2019. The ratios for the selected maternal health indicators indicate the existence of inequalities that favour women with higher levels of education. Moreover, there was a decrease in inequalities between women with no education and those with higher levels of education from 2008 to 2019 as shown by the decrease in ratios.

**Table 2:** Maternal health indicators by maternal education in 2008, 2013 and 2019.

		Maternal education					
Health indicator	Survey year	None	Primary	Secondary	Higher	Total	Ratio
4+ ANC visits	2008	64.2	75.2	83.2	97.2	68.1	1.51
	2013	85.9	86.8	91.6	97.2	87.3	1.13
	2019	88.7	89.1	90.6	93.8	89.5	1.06
Skilled ANC	2008	84.5	92.7	94.6	98.5	86.9	1.17
	2013	96.3	98.0	99.3	98.7	97.1	1.02
	2019	97.6	98.3	98.4	97.6	97.9	1.00
Facility births	2008	20.5	34.5	44.9	70.7	25.3	3.45
	2013	49.9	58.2	71.1	87.6	54.9	1.76
	2019	79.6	84.3	89.1	95.3	83.4	1.20
SBA	2008	35.7	55.9	70.9	94.4	42.4	2.64
	2013	54.2	63.0	78.4	90.8	59.7	1.68
	2019	83.3	86.7	93.2	97.3	86.9	1.17

**Note:** 4+ ANC visits = four or more antenatal care visits; skilled ANC = skilled antenatal care provider; facility births = births delivered in a facility; SBA = births assisted by a skilled birth attendant.

Table 3 examines the prevalence of maternal healthcare use as well as the wealth-based inequality ratios for the selected maternal health indicators. The use of maternal health services increased with socioeconomic status, where there was a higher use of these services among women from the richest households. In terms of the ratios, the findings showed that inequalities favoured women from the richest households. The ratios showed a decline between 2008 and 2019, indicating a decrease in pro-rich maternal health inequalities.

**Table 3:** Maternal health indicators by household wealth quintile in 2008, 2013 and 2019.

		Household wealth						
Health indicator	Survey year	Poorest	Poorer	Middle	Rich	Richest	Total	Ratio
4+ ANC visits	2008	59.1	62.5	66.1	70.8	87.2	68.1	1.48
	2013	83.9	86.3	87.2	87.8	92.5	87.3	1.10
	2019	88.1	90.9	90.8	92.0	84.6	89.5	0.96
Skilled ANC	2008	82.1	83.2	85.9	89.4	96.1	86.9	1.17
	2013	96.0	96.7	96.7	98.1	98.3	97.1	1.02
	2019	98.3	98.5	99.0	98.5	94.9	97.9	0.97
Facility births	2008	17.4	21.8	23.9	28.4	40.5	25.3	2.33
	2013	48.9	50.3	49.7	60.6	71.0	54.9	1.45
	2019	78.6	80.5	83.6	86.1	91.5	83.4	1.16
SBA	2008	28.1	35.4	38.6	49.0	71.4	42.4	2.54
	2013	50.9	52.0	53.2	67.4	83.7	59.7	1.64
	2019	82.3	82.4	86.1	91.8	95.9	86.9	1.17

**Note:** 4+ ANC visits = four or more antenatal care visits; skilled ANC = skilled antenatal care provider; facility births = births delivered in a facility; SBA = births assisted by a skilled birth attendant.

Table 4 shows the prevalence of maternal healthcare use by urban-rural residence and ratios of urban to rural inequalities. The use of maternal health services was higher among women from urban areas than those from rural areas, except for the use of antenatal services in 2019. The ratios for antenatal services in 2019 indicated that inequalities slightly favoured women from rural areas. In general, the ratios in 2008 and 2013 showed that inequalities favoured women from urban areas for all indicators.

**Table 4:** Maternal health indicators by place of residence in 2008, 2013 and 2019.

		Place of residence			
Health indicator	Survey year	Urban	Rural	Total	Ratio
4+ ANC visits	2008	81.6	62.8	68.1	1.30
	2013	91.0	85.8	87.3	1.06
	2019	89.0	89.7	89.5	0.99
Skilled ANC	2008	93.9	84.1	86.9	1.12
	2013	98.2	96.7	97.1	1.02
	2019	97.0	98.5	97.9	0.98
Facility births	2008	40.6	19.6	25.3	2.07
	2013	69.0	50.1	54.9	1.38
	2019	88.9	80.5	83.4	1.10
SBA	2008	66.9	33.2	42.4	2.02
	2013	78.9	53.2	59.7	1.48
	2019	94.1	83.1	86.9	1.13

**Note:** 4+ ANC visits = four or more antenatal care visits; skilled ANC = skilled antenatal care provider; facility births = births delivered in a facility; SBA = births assisted by a skilled birth attendant.

### Concentration curves

**Fig 1:**
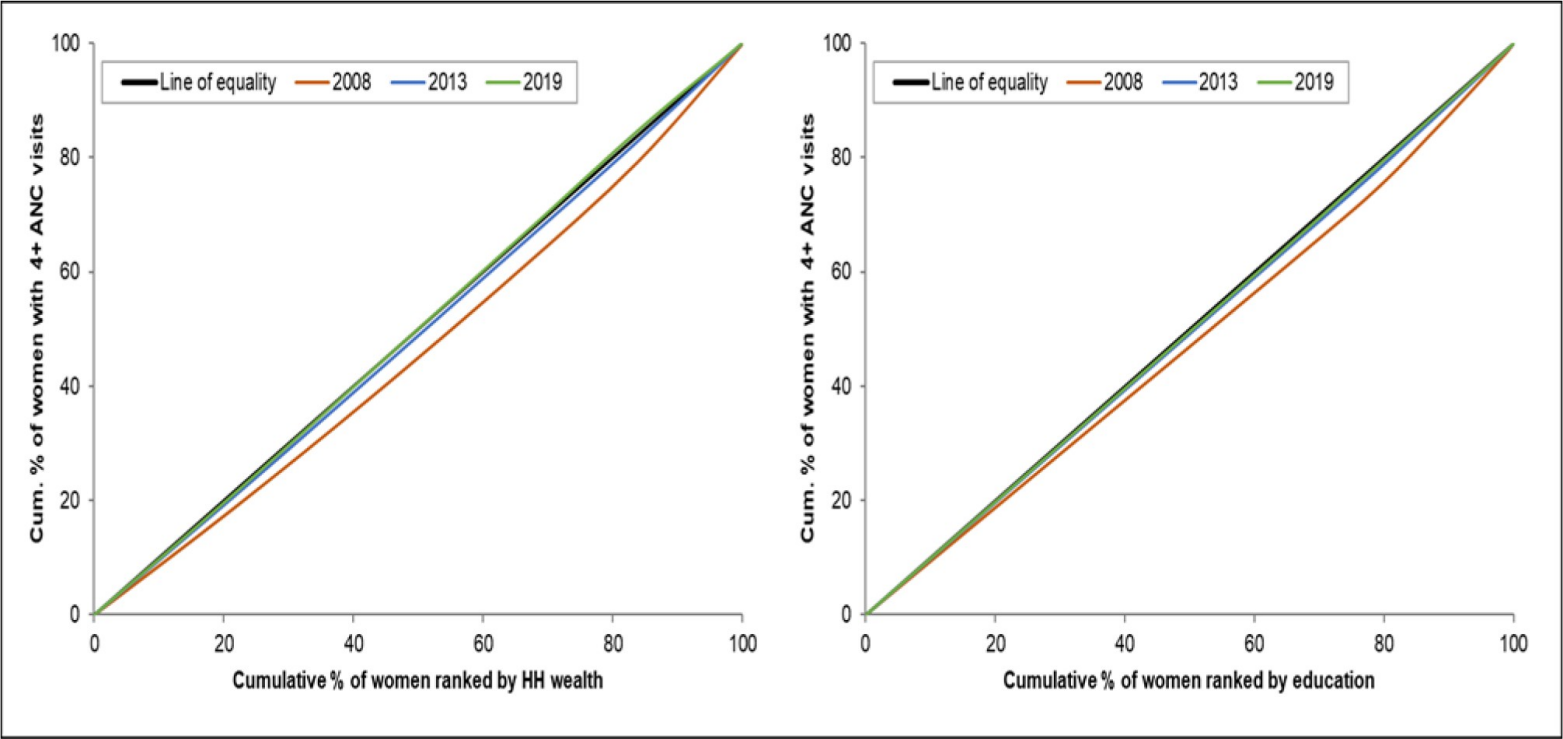
Concentration curves showing wealth-and-education-based inequality in the use of four or more antenatal visits in Sierra Leone, 2008, 2013 and 2019.

**Fig 2:**
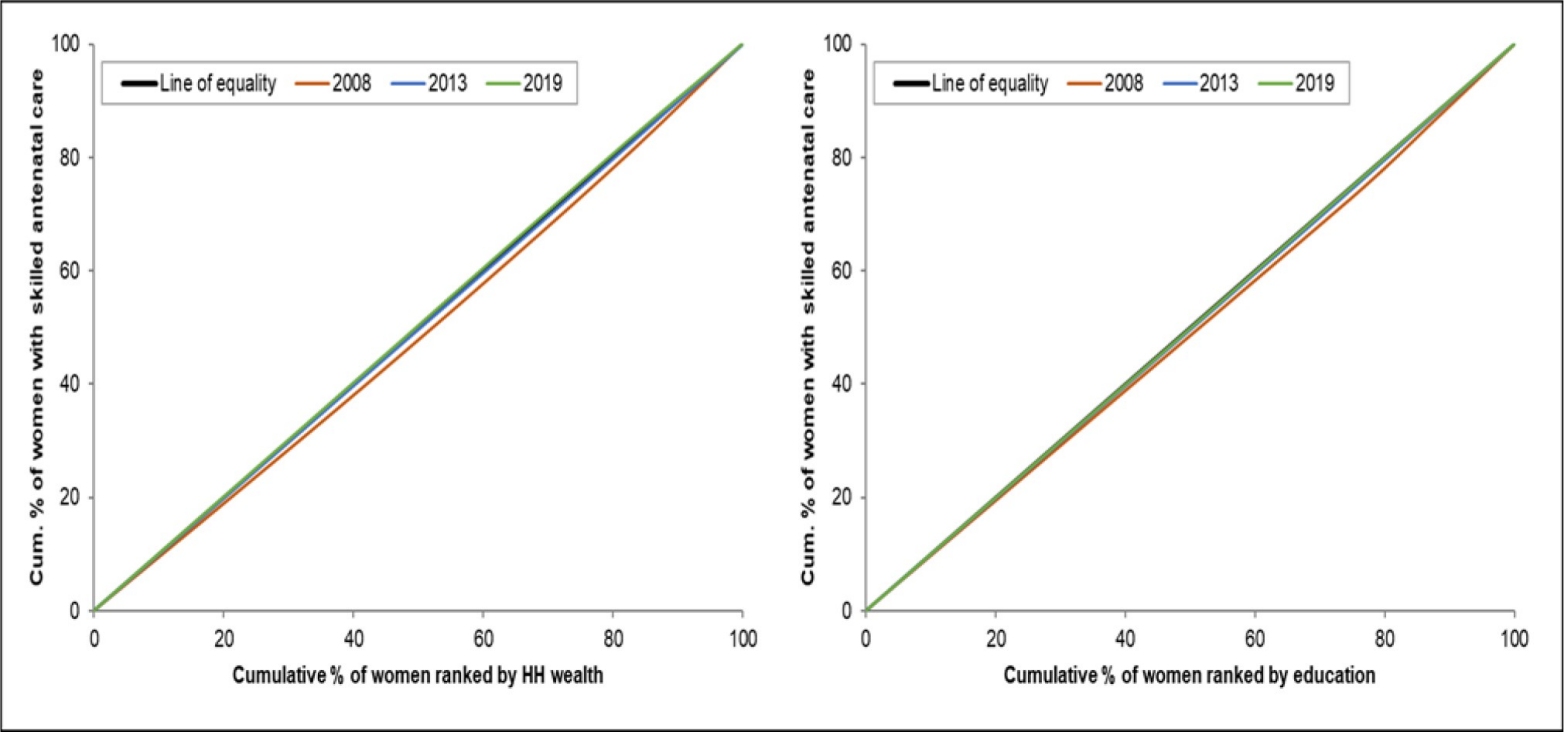
Concentration curves showing wealth-and-education-based inequality in the use of skilled antenatal services in Sierra Leone, 2008, 2013 and 2019.

**Fig 3:**
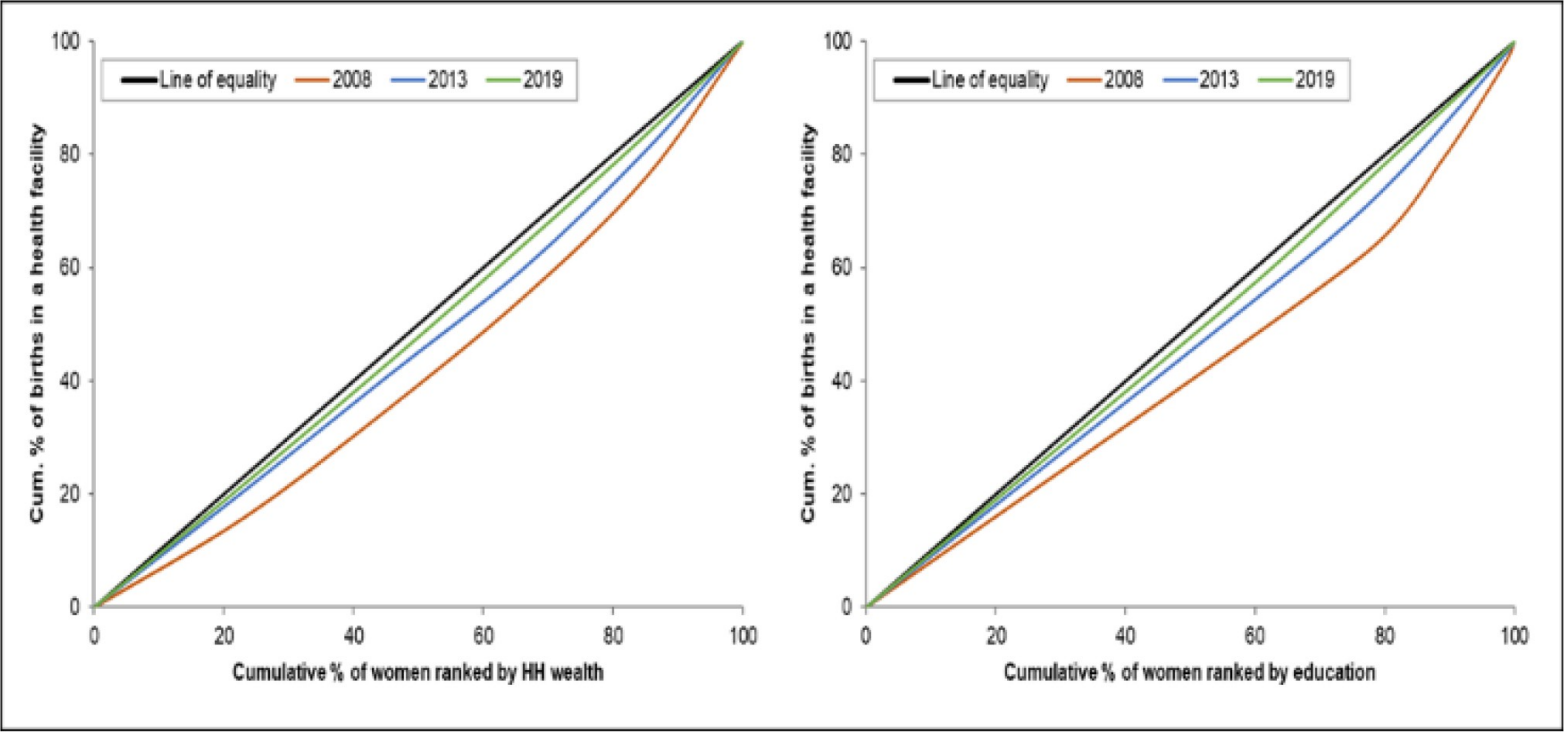
Concentration curves showing wealth-and-education-based inequality in the use of health facilities for delivery in Sierra Leone, 2008, 2013 and 2019.

**Fig 4:**
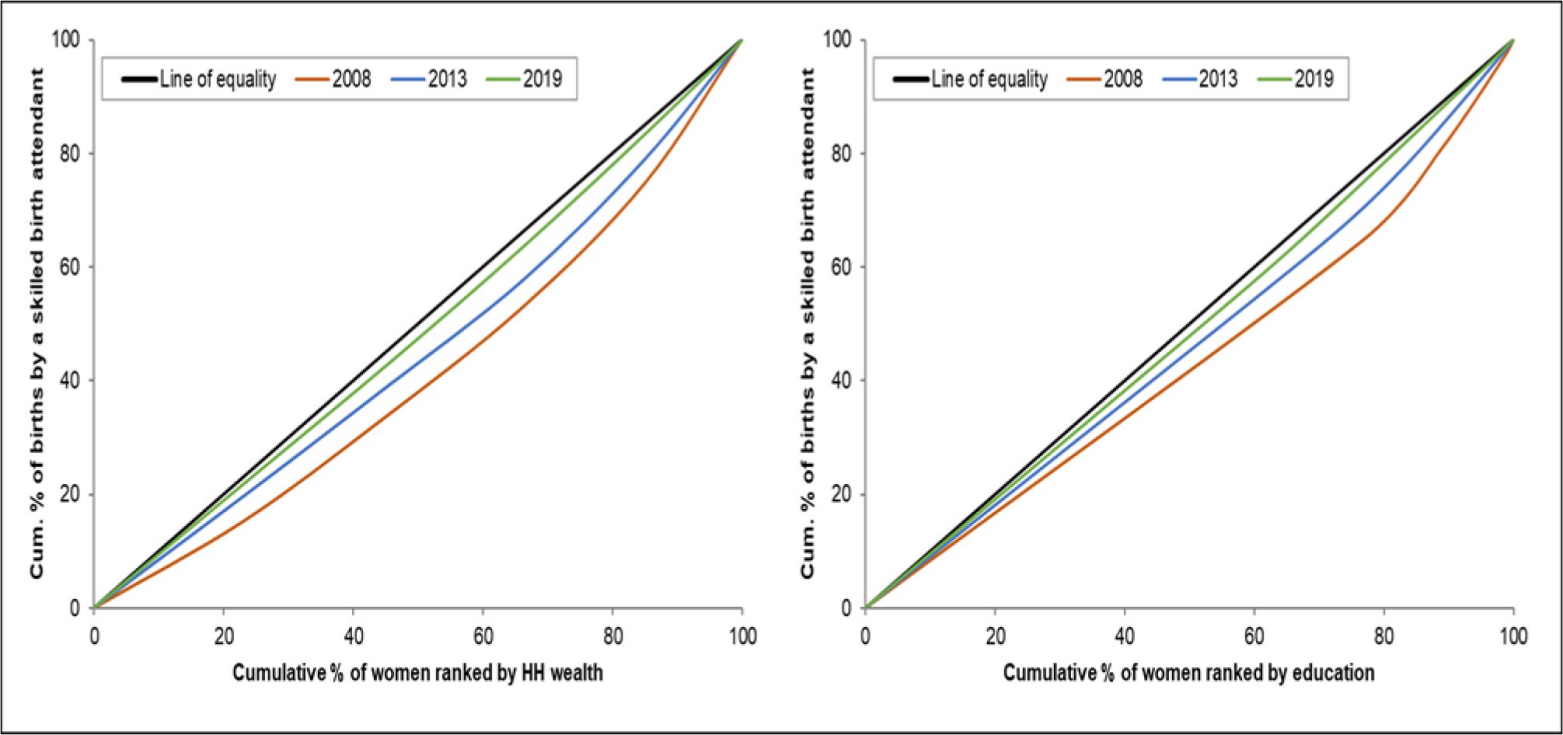
Concentration curves showing wealth-and-education-based inequality in the use of skilled birth attendants in Sierra Leone, 2008, 2013 and 2019.

The concentration curves show that there are inequalities in the use of maternal health services favouring those with a higher socioeconomic position (women with higher levels of education and women from the richest households). There is a higher use of maternal health interventions by wealthier women than by poorer women (Figs 1–4). The inequalities decreased over time, as portrayed by the narrowing of the curves, particularly in the use of antenatal services. Figs 1 and 2 shows that the inequality gap has decreased over time. Moreover, by 2013 and 2019, the inequality gap had almost closed in terms of the use of antenatal services. Furthermore, the findings show that there is high inequality in the use of health facilities for delivery, and skilled birth attendants, as shown by the wide curves.

### Concentration indices

The wealth-based and maternal-education-based concentration indices show that there have been improvements (as shown by the decreasing levels over time) in inequality in the use of maternal healthcare use for about eleven years, from 2008–2019 (Tables 5 and 6). The biggest decrease in the concentration index was for the use of births assisted by a skilled birth attendant, which decreased from 0.330 to 0.113 for wealth-based inequalities and 0.230 to 0.095 for education-based inequalities (Tables 5 and 6). Conversely, there was high inequality in the use of delivery care services (births delivered in a facility and births assisted by a skilled birth attendant), in both 2008 and 2013.

**Table 5:** Concentration indices showing wealth-based inequality in the use of maternal health services in Sierra Leone, 2008, 2013 and 2019.

Health indicator	Survey year	Conc. Index (CI)	SE (CI)	95% CI	
				Low	High
4+ ANC visits	2008	0.197*	0.023	0.152	0.243
	2013	0.052*	0.013	0.026	0.077
	2019	0.016	0.013	-0.010	0.042
Skilled ANC	2008	0.104*	0.017	0.070	0.138
	2013	0.022*	0.005	0.012	0.032
	2019	-0.007	0.007	-0.021	0.008
Facility births	2008	0.202*	0.024	0.155	0.249
	2013	0.196*	0.024	0.148	0.244
	2019	0.109*	0.019	0.072	0.147
SBA	2008	0.330*	0.026	0.279	0.382
	2013	0.250*	0.023	0.206	0.295
	2019	0.113*	0.016	0.081	0.145

**Note**: * = p<0.001

** = p<0.01; 4+ ANC visits = four or more antenatal care visits; skilled ANC = skilled antenatal care provider; facility births = births delivered in a facility; SBA = births assisted by a skilled birth attendant.

**Table 6:** Concentration indices showing education-based inequality in the use of maternal health services in Sierra Leone, 2008, 2013 and 2019.

Health indicator	Survey year	Conc. Index (CI)	SE (CI)	95% CI	
				Low	High
4+ ANC visits	2008	0.128*	0.015	0.099	0.157
	2013	0.038*	0.009	0.021	0.056
	2019	0.021**	0.008	0.005	0.038
Skilled ANC	2008	0.070*	0.010	0.050	0.090
	2013	0.026*	0.004	0.018	0.034
	2019	0.010**	0.004	0.003	0.017
Facility births	2008	0.170*	0.015	0.141	0.200
	2013	0.160*	0.013	0.135	0.185
	2019	0.097*	0.012	0.072	0.121
SBA	2008	0.230*	0.016	0.199	0.261
	2013	0.179*	0.013	0.155	0.204
	2019	0.095*	0.012	0.071	0.118

**Note**: * = p<0.001

** = p<0.01; 4+ ANC visits = four or more antenatal care visits; skilled ANC = skilled antenatal care provider; facility births = births delivered in a facility; SBA = births assisted by a skilled birth attendant.

## Discussion

This study aimed to explore health inequalities in maternal healthcare in Sierra Leone. The findings show that considerable progress has been made in the use of maternal health services; the measures employed in the study show that inequalities in maternal healthcare use have declined since 2008. Our findings suggest that maternal health inequalities favour women from wealthy households, educated women, as well as women from urban areas. This could be because women with better socioeconomic status (wealthy households and higher education) tend to live in urban areas and can better pay for use and the available health services compared to their counterparts [36,37]. Moreover, improvements in wealth and education-based inequality were evident in 2013 and 2019 for the use of antenatal services. Although inequality declined over time, the use of delivery care services remained highly unequal. Our findings are similar to other studies which found substantial inequalities in the use of delivery services; similar studies show that inequalities in delivery care services tend to favour wealthier and more educated women [3,34,37,38]. A study in Sierra Leone also found that maternal education made a considerable contribution to inequalities in institutional delivery [16]. The cultural aspects of the population as well as their perceptions of modern medicine and related health provision are critical in understanding the use of maternal healthcare services [16,39].

Additionally, the findings show that there is some degree of inequality favouring populations in urban areas; this supports the literature which argues women in urban areas tend to have greater access to maternal health services compared to women in rural areas [40,41]. These findings speak to the rural-urban gap in the provision of healthcare services between rural and urban areas as well as the related barriers, such as costs and distance, disproportionally faced by women in rural areas [13–15,42]. The introduction of the FHCI and the removal of user fees might have contributed to the increase in the use of maternal health services as well as the reduction of inequality in the use of these services. Witter and colleagues have conducted numerous studies, which monitored and evaluated the main pillars of the FHCI concerning how these pillars have been implemented on the ground [43,44]. The authors argue that the use of maternal health services in the country has increased, and this increase could be attributed to the implementation of the FHCI [43,45,46]. It is difficult to pinpoint the exact contribution of the FHCI to increasing the use of health services since some of these health services had high uptake rates before the implementation of the FHCI [46]. Although the FHCI may have contributed to an increase in the use of maternal healthcare services, there is still some level of inequality that exists in the use of maternal healthcare services in the period highlighted in this study. A comprehensive analysis of the impact of this initiative (and other related initiatives) on the use of maternal health services and related inequalities in the country will be important for future research.

## Strengths and limitations

The main strength of this study is that we used nationally representative datasets from three collection periods to better estimate inequalities in maternal healthcare use. The study uses cross-sectional data, as such, the data cannot serve as the basis for establishing causality among variables. There may be recall bias because of the longer recall time, where respondents are required to report on past occurrences of the use of certain healthcare services.

## Conclusion

Our findings show that despite efforts by the government to increase the use of maternal healthcare services among women with a lower socioeconomic status, the use of these services remains favourable to those with a higher socioeconomic status. To ensure balance among the different socioeconomic groups, policy initiatives need to prioritise women with lower socioeconomic status (those with the most unequal maternal health services) through projects aimed at reducing poverty and increasing their educational levels, especially among women from rural areas. Moreover, further studies are necessary to study the specific impact of the FHCI and similar initiatives on the use of maternal healthcare services in the country, and what impact these initiatives have had on the reduction of health inequalities.

## Abbreviations

DHS: Demographic and Health Survey
FHCI: Free Health Care Initiative
MMR: Maternal Mortality Ratios
MDGs: Millennium Development Goals
PCA: Principal Components Analysis
SBA: Skilled Birth Attendant
SDGs: Sustainable Development Goals
